# Memory for facial expression is influenced by the background music playing during study

**DOI:** 10.2478/v10053-008-0118-9

**Published:** 2012-08-21

**Authors:** Michael R. Woloszyn, Laura Ewert

**Affiliations:** Department of Psychology, Thompson Rivers University, Kamloops, British Columbia, Canada

**Keywords:** music, emotion, memory, facial expression

## Abstract

The effect of the emotional quality of study-phase background music on subsequent recall for happy and sad facial expressions was investigated. Undergraduates (*N* = 48) viewed a series of line drawings depicting a happy or sad child in a variety of environments that were each accompanied by happy or sad music. Although memory for faces was very accurate, emotionally incongruent background music biased subsequent memory for facial expressions, increasing the likelihood that happy faces were recalled as sad when sad music was previously heard, and that sad faces were recalled as happy when happy music was previously heard. Overall, the results indicated that when recalling a scene, the emotional tone is set by an integration of stimulus features from several modalities.

## Introduction

Moviemakers have long known about the effect that a stirring piece of music can have on one’s perception of a scene. Testifying to the power of this phenomenon is the fact that the use of music to convey information and set a mood for a film predates the use of sound in a movie altogether (Cook, 1990). Depending on the emotional quality of the melody, music can be used to bias a viewer’s expectations of plot development (Vitouch, 2001), to influence the perceived emotional content of otherwise valence-neutral film clips (Eldar, Ganor, Admon, Bleich, & Hendler, 2007) and static pictures (Spreckelmeyer, Kutas, Urbach, Altenmüller, & Münte, 2006), or possibly to generate an emotional response from the observer (Goldstein, 1980; Steinbeis, Koelsch, & Sloboda, 2006), although this last assertion is not without its critics. As Koneni (2008) somewhat controversially pointed out, at least some readers (and researchers) appear to confuse the ability of a piece of music to represent an emotion with that same piece evoking one (e.g., Sloboda & Lehmann, 2001), and that the available evidence in favour of short snippets of music leading to a change in the emotional state of the participant is less prevalent than one might think (see Koneni, 2008, for a detailed review). Despite these concerns, however, most theorists and researchers would agree that music as a stimulus has the ability to represent and, at least under certain conditions, to evoke measureable emotional responses in listeners in the form of changes in skin conductance, respiration, heart rate, or self-report measures (Baumgartner, Esslen, & Jäncke, 2006; Kreutz, Ott, Teichmann, Osawa, & Vaitl, 2008).

Just as music can alter expectations and interpretations of a visual scene, the causal connection between music and cognition (e.g., evaluations) of a situation appears to work both ways, in that the perceived emotional tone of the accompanying music can be influenced by visual aspects of the scene or situation, such as the facial expression of the performer (Thompson, Graham, & Russo, 2005). Although there is a large body of literature on the topic of encoding specificity (Tulving & Thompson, 1973), and the presence versus absence of encoding cues at retrieval (Tulving & Osler, 1968; Tulving & Pearlstone, 1966), little work appears to have taken place examining the effects of the emotional tone of background music on memory for visual emotional stimuli. This connection forms the focus of the work presented here. Specifically, we were interested in knowing whether or not the presence of emotion-conveying background music while studying pictures would influencesubsequent memory for the facial expressions of characters depicted in those pictures. Generally, the perception of facial expression involves a number of diverse and interactive brain regions that make use of both the features in the face itself and of any auxiliary emotion-conveying contextual information (Adolphs, 2006) in arriving at a perceived emotional tone being conveyed in a scene. If it is the case that any aspect of context could potentially contribute to the perception of a facial expression, then the emotional tone set by the music paired with a picture should influence recall of emotional aspects of the picture when subsequently tested, by biasing responses toward the mood conveyed by the music. That is, if during encoding a photo with an individual displaying a particular facial expression (e.g., smiling) appears in conjunction with a musical excerpt that conveys conflicting emotional information (sadness), will the subsequent recall of the emotional facial expression be influenced by the emotional musical context during encoding?

The current study was designed to investigate the extent to which brief happy or sad melodies heard while viewing a series of line drawings of a child in a variety of emotionally neutral environments will affect later recall for the child’s emotional facial expression. Several possible outcomes are possible.

For example, it is possible that a given facial expression’s distinctiveness predicts its subsequent recall because *distinctiveness*, in one form or another, influences memory (Murdock, 1960; Neath, Brown, McCormack, Chater, & Freeman, 2006; von Restorff, 1933). Specifically, it is possible that the juxtaposition of a facial expression with valence-conflicting music during encoding (e.g., a crying child with happy background music), might actually enhance memory for this facial expression, due to the greater distinctiveness of this stimulus combination relative to either concordant audio/visual pairs, or to a baseline consisting of a visual scene with no accompanying music.

Alternatively, it is possible that the emotion conveyed by the music paired with a picture increases recall of a facial expression corresponding to that particular musical emotion. This influence of music on later recall should be observed relative to baseline pictures without music. Musical emotion could either increase the accuracy of the memory for an emotionally concordant facial expression, or bias the perception of the emotional facial expression to conform to the musical valence present during encoding. In the latter case, the same bias should be found for discordant pairs. To start with an increased accuracy, researchers have long known about the beneficial effects of either prior or concomitant exposure to musical stimuli on certain cognitive tasks such as spatial-temporal ability (Rauscher, Shaw, & Ky, 1993), as well as on memory for text (Rainey & Larsen, 2002; Wallace, 1994) or pictures (Carr & Rickard, 2010). So, it is possible that the musical excerpts benefit the participants’ later retrieval. One caveat for this prediction, however, is the fact that in nearly all of the work mentioned above, participants were required to listen to music for a significantly longer period of time than participants in our study.

With reference to the potential biasing effect of music on perception, recent work has shown that participants’ judgments of the facial expression (happy, sad, neutral) of a person in a photograph were primed by prior exposure to brief happy or sad melodies. For example, Logeswaran and Bhattacharya (2009) found that recognition of the facial expressions were highly accurate, but more importantly, that the judgment of how happy or sad the face was had been influenced by prior music, so that happy music led to higher happiness ratings and lower sadness ratings for the happy and sad faces, respectively. Given this, it is possible that pairing emotionally concordant music and facial expressions might result in greater “accuracy” than if the music and facial expression are in conflict with regard to the emotion being conveyed, or if the images appear with no music accompaniment. This influence of concordant music could be due either to a biasing influence of music on memory (Logeswaran, & Bhattacharya, 2009) or to a perceptual integrative process involving a combination of the emotional quality of the face and the music (Thompson, Russo, & Quinto, 2008).

A third possibility, based on findings from recent work looking into the neural correlates of memory for emotionally charged stimuli, is that a different pattern of response accuracies might arise depending on whether the music paired with a given stimulus is happy or sad. A wealth of research has confirmed that in general, relative to the neutral condition, any emotional context, be it positive or negative, can enhance retrieval (for an overview, see Hamann, 2001, or LeDoux, 2000). More recently, work has narrowed down the specific brain areas that might mediate these differences. Smith, Henson, Dolan, and Rugg (2004), for example, found that different patterns of neural activity mediate the retrieval of memory for images paired with contexts consisting of a variety of valences (positive, neutral, or negative). Specifically, they discovered that the ability to recognize previously presented negative stimuli was associated with heightened activity in specific regions including “the left fusiform, left middle occipital gyrus, bilateral middle temporal gyri, bilateral cuneus, and left medial frontal gyrus” (Smith et al., 2004, p. 872). Additionally, they have found overall superior re-cognition with positive-valence items in comparison with negative or neutral contexts (for similar results, see Erk et al., 2003), and that these differences appear to be linked to differential activity between valence conditions in the left and right and posterior cortical region (cf. also Maratos & Rugg, 2001), frontal regions (cf. Schmidt & Trainor, 2001), and the amygdala (cf. also Hamann, Ely, Grafton, & Kilts, 1999).

Given the wealth of data showing that stimuli with an emotional component can influence retrieval differently depending on the qualityof the emotion being induced or represented, it stands to reason that one might expect superior rates of recall for faces paired with emotionally positive melodies relative to faces paired with either negative-valence music, or no music, possibly as a result of increased arousal for those stimuli. Such a notion forms the basis of the arousal-mood hypothesis, which states that listening to music of different valences will lead to dif-ferential performance on various cognitive tasks (Husain, Thompson, & Schellenberg, 2002). If the melodies employed in the study reported here do give rise to a quantifiable level of arousal in participants, then one would expect to see an overall main effect of music type (happy vs. sad) on subsequent retrieval, but little in the way of an interaction.

We cannot, however, ignore the fact that the primary focus of this research is on the retrieval of facial expressions as opposed to measuring the influence of the background melodies on arousal or mood. Here again, a wealth of research exists pertaining to the issue of memory for faces and facial expressions, from both the phenomenological and neurological domains. In particular, a cluster of interconnected regions well-known to be involved in emotion (e.g., amygdala, orbito-frontal cortex, basal ganglia) appear to be responsible for the encoding and recognition of emotional facial expressions (Adolphs, 2002). Furthermore, there is evidence of differential hemispheric activity depending on the valence of the facial expression being either positive (happy) or negative (angry, sad), with increased left hemisphere activity for the former, right hemisphere activity for the latter (see Shimamura, Ross, & Bennet, 2006); similar asymmetric responding appears when the stimuli consist of a multidimensional combination of facial expressions and voice depicting either happiness or fear (Pourtois, de Gelder, Bol, & Crommelinck, 2005). Additionally, evidence indicates that faces with positive emotional expressions are associated with superior retrieval relative to angry (D’Argembeau & Van der Linden, 2007), or neutral (Shimamura et al., 2006) facial expressions, and that this effect appears to be due to the angry faces having a detrimental effect on memory, as opposed to the happy expressions enhancing it (D’Argembeau & Van der Linden, 2011). If such an effect extends to any facial expression with negative valence (fear, sadness), then this leads to the prediction that previously studied happy faces should show superior recall at test relative to the sad faces. In summary, based on the data indicating superior processing of information in a happy-valence musical context, and on the fact that positive facial expressions tend to be associated with superior memory over the negative ones, one might expect to see happy face-happy music trial types being better recalled than happy face-sad music, or sad face-happy music, with sad face-sad music being associated with the lowest proportion of correct values.

## Method

### Participants

A total of 48 Thompson Rivers University undergraduate students (16 male and 32 female) received partial course credit in exchange for their participation. All participants reported normal or corrected-to-normal eyesight, and no hearing deficits.

### Materials

**Figure 1.**
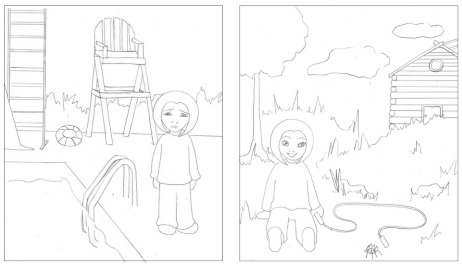
Examples of study stimuli.

A series of 42 simple line drawings (created by Laura Ewert) were employed in the study, each depicting a child either laughing or frowning in various recognizable environments and situations (see Figure 1, for examples). The use of line drawings allowed for the control of potential salience differences among the scenes that might appear if real photos were employed. Also, creating a series of distinctly different line drawings allowed the same facial expression and androgynous child to be used repeatedly without having recognition cues such as physical attractiveness and familiarity potentially enhancing memory for the character. Pilot work confirmed that the backgrounds were neutral with regard to biasing viewers to respond “happy” or “sad,” by allowing participants to view 60 sample drawings featuring the child without a facial expression. For each, the participants were asked to select which emotional expression (happy or sad) would be best suited for the child’s reaction to the setting depicted in each image. The 42 background images associated with no significant response tendencies toward either “happy” or “sad” were selected for use in the study reported here.

From this collection of picture stimuli, six stimulus types (each type consisting of seven different scenes) were created by independently varying the facial expression of the character in each photo (happy/sad) and the accompanying music (happy/sad/no music), resulting in a 2 × 3 factorial design. Two groups of participants were used to counterbalance the facial expressions across pictures, so that if Group 1 saw a particular picture with a face of one expression (e.g., happy), Group 2 saw the same picture depicting the opposite facial expression (e.g., sad).

Many previous studies on the perceptual influences of visual and auditory aspects of a scene have used well known music that may already be familiar to the listener and thus might either elicit pre-existing emotionally charged associates when played, or might itself be differentially memorable to participants (Eschrich, Münte, & Altenmüller, 2008). To avoid this potential confound, music clips that conveyed sadness or happiness were obtained from a standardized set of emotional music excerpts constructed for this purpose (Vieillard et al., 2008). Each clip consisted of a digital piano playing a short excerpt of a song that was rated as either “happy” or “sad” by listeners. In Vieillard et al’s (2008) original set, clips varied widely in their duration. To avoid this, we edited the clips to each be 7 s in length, fading at the end. This also served a second purpose of providing the participant with a warning that the picture being viewed was about to change. The pieces varied in terms of their pitch register, time signatures, and key from one to the next. The sole factor (aside from whether the piece was in a major or minor mode) that seemed to systematically differentiate the “happy” from “sad” pieces was that the sad excerpts tended to have a somewhat slower tempo relative to the happy excerpts. Generally, the happy pieces tended to fall in the Allegretto-Allegro range (~110-140 beats per minute [bpm]), whereas the sad pieces were more in the Adagio-Andante range (~66-110 bpm).

### Procedure

Participants were told that they would see a series of line drawings of a person in a variety of environments with some background music, and were asked to study the pictures as best they could, because their memory for the images would later be tested. For the study phase, the lights were turned off and each picture was presented on an overhead screen with its accompanying melody for 7 s, followed by a blank slide for 2 s. Upon conclusion of the study phase, the 42 drawings were subsequently represented with the face of the main character blanked out, and no accompanying background music. For each blank face, participants were required to indicate whether they recalled previously seeing the child as being “happy” or “sad” by circling the appropriate word on a response sheet. In the event that they could not remember which facial expression they saw, they were told to guess.

## Results

**Figure 2.**
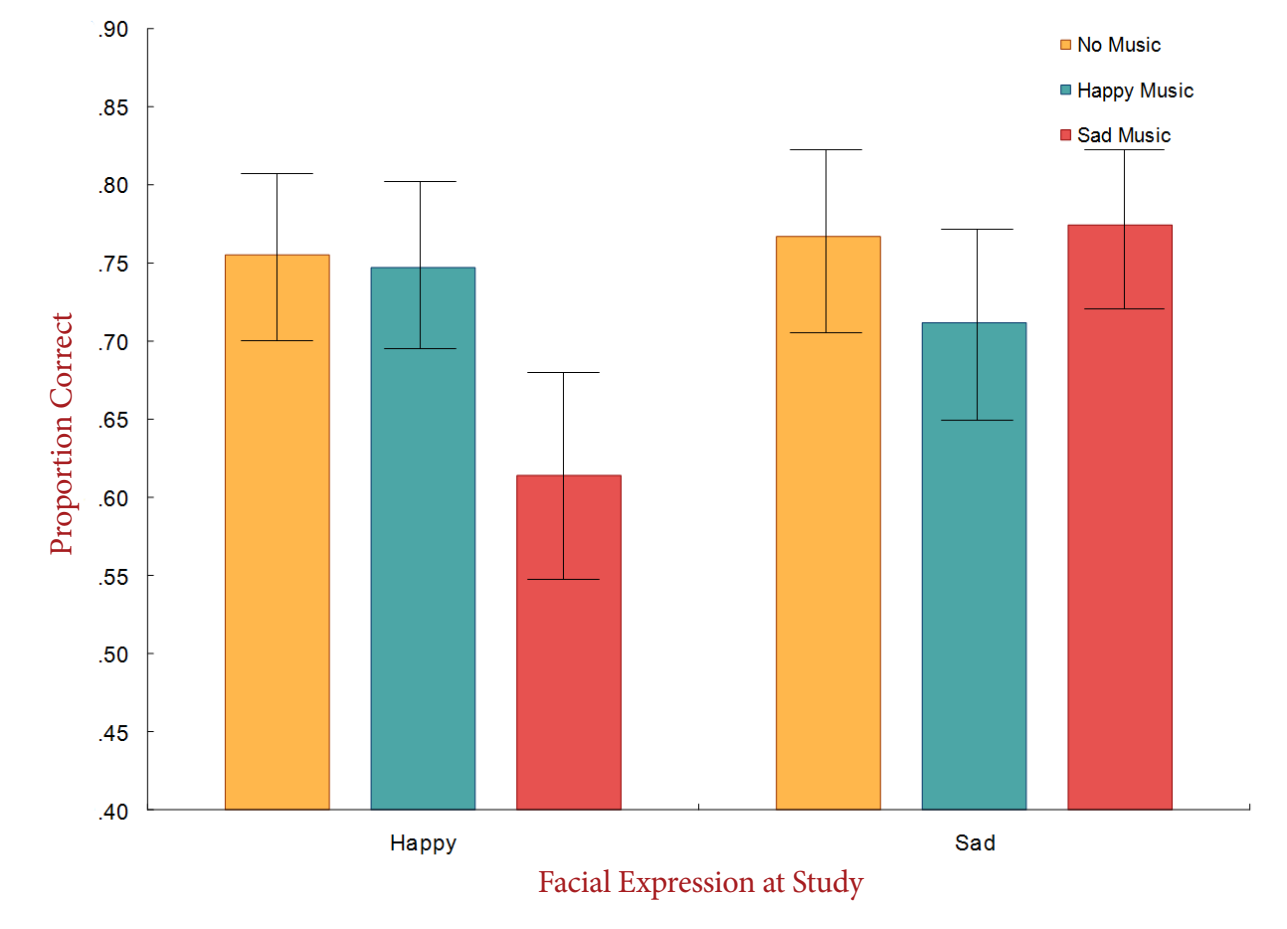
Proportion correct means and standard errors for all conditions (collapsed across order).

Participant responses were transformed into proportion correct for each trial type, and submitted to a 2 (facial expression: happy, sad) × 3 (musical accompaniment: no music, happy music, sad music) × 2 (presentation order) mixed-design ANOVA. Figure 2 depicts the means and 95% confidence intervals for each condition, collapsed across order (the presentation order variable did not enter into any significant effect or interaction).

There was a small but significant main effect of facial expression at study, with slightly lower accuracies for happy (*M* = .71, *SE* = .023) relative to sad (*M* = .75, *SE* = .022) faces, *F*(1, 46) = 4.18, *p* = .047, η_p_^2^= .083. A main effect also occurred for music type, *F*(2, 92) = 4.43, *p* = .015, η_p_^2^= .088. Generally, it appears that a decreasing trend in recall accuracy occurred when moving from no music (*M* = .761, *SE* = .024) to happy (*M* = .73, *SE* = .022) and sad (*M* = .694, *SE* = .024) music but Bonferroni-adjusted comparisons confirmed that the sole difference among the means occurred between the no-music and sad-music conditions (*SE* = .023, *p* = .017).

Finally, a significant interaction occurred between Facial Expression and Musical Accompaniment, *F*(2, 94) = 4.42, *p* = .015, η_p_^2^= .163. Specifically, Tukey’s HSD revealed that studied happy faces were more likely to be recalled when happy as opposed to sad music was previously heard (*p* < .05), whereas this increasing pattern did not occur for sad faces (*ns*). In fact, upon visual inspection, it appears that a crossover effect occurred in the data, in that concordant faces and music (happy-happy or sad-sad) gave rise to somewhat higher mean accuracies than discordant faces and music (happy-sad or sad-happy). To further explore whether this pattern is significant, a trends analysis was carried out to investigate whether or not said cross-over effect is significant, by assigning appropriate weights for the comparison reflecting a pattern in which going from happy to sad music should lead to a decreasing trend in accuracy of recall of happy faces, but an increasing trend in accuracy of recall of sad faces. The analysis for this pattern revealed that the obtained data significantly matched the prediction, *F*(1, 96) = 18.02, *MSE* = .0253, *p* < .001, implying that, indeed, the pattern of effect of music depends on the facial expression participants had previously viewed, with a significant crossover occurring.

## Discussion

The results of the present study support the hypothesis that the valence of music presented during study of visual stimuli can affect subsequent recall for facial expressions. This is apparent in the obtained pattern of errors, in that happy faces paired with sad music resulted in fewer “happy” responses relative to the same faces being previously paired with happy music (and vice versa for sad faces). Additionally, it is interesting that the obtained pattern of results does not conform to what one would expect based on a combination of the bulk of research that indicates an advantage of happy over sad music and faces described in the Introduction. Specifically, it appeared that contrary to the expectation of happy music and faces in conjunction being associated with the highest accuracies, and sad music and faces being associated with the lowest, we found that when the two modalities were in concert with one another, the accuracies were higher than when they were in conflict. Furthermore, such a pattern would not be expected based on a mood-arousal mechanism (Husain et al, 2002) which would predict an overall improvement in memory for faces previously paired with happy but not sad music. Such a main effect did occur. However, the interaction does not fit with Husain et al.’s explanation, in that the happy music only produced a significant benefit when a happy face was viewed. As a result, the question remains as to what mechanism might be responsible for this phenomenon.

It is, first of all, likely that this phenomenon is a result of the processing taking place during study, as opposed to some aspect of the test conditions giving rise to the response tendency. The main reason for this supposition is due to the fact that at test there were no overt emotionally-charged stimuli available to participants. The facial expressions were blank, and no background music was playing. The sole cue at their disposal, the scene itself, was previously found to be emotionally neutral with regard to biasing participants toward one or the other facial expression. If the resulting response is a consequence of the scene itself re-instantiating a memory representation of both the music and facial expression that occurred at study, then the only way such a pattern of responding could be obtained would require the memory for the background music to be more accurate than the memory for the facial expression, resulting in the former “overriding” the latter at test. That is, participants, when viewing the scene, would have a better record of the music playing than the facial expression being made in memory, and would bias their responses to fit that representation. This account is problematic, however, due to the fact that such a bias did not seem to influence responding when the music and facial expression were representing the same emotion (i.e., both happy or both sad), in that responses for those conditions were indistinguishable from the condition where no music appeared. On the other hand, if, during study, the memory representation is laid down as a result of an overall average impression resulting from an integration of facial expression and background music, then the predicted accuracy would be that which was obtained, in that the “averaging” should give rise to a drop in accuracy for scenes where the facial expression and music are contradictory, whereas no such change should occur when they are in concert. As for specific mechanisms that took place during study, several alternatives come to mind.

One possibility is that the music during study gave rise to an emotional experience, which itself was later confused with the portrayed facial expression. Such a mechanism is corroborated by a large body of evidence in studies of encoding specificity and mood-dependent memory showing that an emotional state invoked during study can be recruited to serve as a recall cue (see Lewis & Critchley, 2003, for a summary). Mitigating against this is the fact that the melodies appeared for a very brief period, were highly impoverished (consisting of a single stream of notes played on a digital piano with no expressiveness or harmonic lines), and were intermingled with one another (so that in many instances, the melodies alternated from happy to sad to happy again), meaning any influence on the mood of the participant, presumably a relatively durable condition being resistant to such minor environmental features, would be small at best, given that the induction of a mood would require time and relatively emotionally rich stimuli (Koneni, 2008).

An alternative mechanism relies not on the emotional experience elicited by the music, but rather on the emotional label portrayed by the music as being later mistakenly used to infer the facial expression. In other words, the clips in our study may have affected memory for facial expressions presented concurrently either by activating a general emotional schema used to represent the scene, or as a result of a source confusion error during recall (Johnson, Hashtroudi, & Lindsay, 1993). To illustrate, according to a schema explanation, a participant might recall a person in a stimulus photo as smiling because the happy melody paired with the scene might cause the information to be encoded in memory as being a “happy” situation. Alternatively, according to a source-confusion explanation, the emotional tone of the background music may later become confused in memory with the facial expression displayed by the character. The study reported here was not designed to test between these two possibilities, and no simple manipulation comes to mind regarding how one might do so. Our results, however, are at least consistent with research demonstrating that music plays a role in the encoding of visual information. Many studies use vocalizations paired with faces for emotion interpretation (e.g., de Gelder & Vroomen, 2000; Thompson et al., 2008); however, the current findings extend the dimension of auditory information that can be integrated with facial expressions to influence recall, possibly occurring at the point of encoding or during memory consolidation. In summary, these findings suggest that the emotional tone conveyed by background music can influence subsequent memory for emotional details of an event, indicating the cross-modal nature of memory for emotional aspects of situations.

Future work will focus on experimentally teasing apart the various explanations entertained above, as well as should address some of the limitations apparent in the current study. For example, we did not examine differences in musical training and their potential influence on the pattern of results obtained. The primary reason for this was due to the focus of the work being squarely on the memory for the faces and their interaction with the mood conveyed by the clips, rather than on the music per se. Although some research exists that supports the notion that musicians possess superior cognitive skills in certain tasks (Moreno et al., 2008; Schellenberg, 2005), and superior memory abilities for certain stimulus types (Jakobson, Cuddy, & Kilgour, 2003), some argue that the superior memory ability is somewhat limited primarily to the auditory realm (Cohen, Evans, Horowitz, & Wolfe, 2011). As a result, any musicians participating in our study would have primarily a memory enhancement for the music that played in a particular scene, but not necessarily what face was presented in that scene. That limitation notwithstanding, though, if it is true that musicians possess superior memory for facial expressions, then one should see primarily the musicians scoring with high accuracy on all trial types. This would have the effect of diluting the data, serving to reduce the size of any effect or interaction. As such, if our sample did contain more than a few highly trained musicians, it would have made it less likely for us to observe the pattern of effects we observed, not more. For now, however, we cannot draw any conclusions about the relative ability of musicians and non musicians in this task.

A second limitation pertains to the somewhat impoverished and unrealistic stimuli employed. In particular, we chose to use line drawings as opposed to more realistic scenes and faces, and employed music that was performed by a computer, lacking any overt expressiveness. In the case of the visual scenes used, as indicated earlier, our decision to do so was in order to maintain control over the quality, amount of detail, presence of the same character, and general similarity of the scenes from one to the next, in an effort to reduce the possibility of any pop-out scenes accidentally giving rise to superior memory that might occur if forced to use realistic photos and people. As a result, we can only speculate whether or not similar effects would be obtained if real faces and scenes were employed, although it should be noted that in favor of the notion that similar results should occur with more realistic stimuli is the fact that brain areas implicated in face recognition respond equally strongly to real and cartoon faces (Kanwisher, Stanley, & Harris, 1999). Again, future work can explore this more fully. As for the musical excerpts, we used the original stimuli designed and recorded by Vieillard et al. (2008), for which the validity data was obtained concerning the emotional quality of the pieces played. To alter their stimuli by adding expressiveness would introduce a source of variability that may have unduly influenced the memorability of the clips, if some were, as a result of the expressiveness added, made to be more or less striking than others.

One other potential limitation pertains to the fact that we did not assess the overall mood of each participant prior to their taking part in the study, something that may have interacted with the results obtained. This limitation is somewhat mitigated, however, by the fact that we ran a within-subjects design, such that any influence of mood would be made somewhat moot by the fact that participants served as their own controls, making overall mood a constant factor across the levels examined for a given participant, and any potential effect of mood a random factor across participants, thus lowering the likelihood of any significant result being obtained. We acknowledge, however, the fact that this is a potential source of between-subjects variability that can be accounted for in future work, which hopefully would strengthen any results obtained.

Future research will, in addition to addressing some of the above limitations, also take steps to try and separate the impact on memory of the emotional quality of the music from the emotion experienced in participants. Our study did not differentiate between the separate influences of the portrayed emotional quality in the music from any emotional experience the music may have given rise to in participants during study. Examining these factors separately would require presenting participants with music that portrays an emotional quality (“happy”), but does not give rise to a corresponding emotional experience, and likewise, a corresponding set of melodies that give rise to a corresponding emotional experience, without portraying said experience. We know of no such stimulus set, and believe it is unlikely that anyone will be able to come up with such a set that would allow us to independently vary the emotion being portrayed and the emotion being induced. Instead, an alternative means of doing this would be to ask participants to rate, while listening to the study materials, the extent to which the stimulus is representing an emotion, and the extent to which the same stimulus is producing an emotional experience. This information can subsequently be used to tease apart the influence of the two factors, by separately entering them as covariates to statistically remove their influence and examine the resulting effect on memory accuracy. If doing so results in a disappearance of the effect on memory scores for one of the covariates but not the other, then it is corroborating evidence that the factor represented by that covariate was what was responsible for the effect in the first place.
